# (Dis)agreement of polymyalgia rheumatica relapse criteria, and prediction of relapse in a retrospective cohort

**DOI:** 10.1186/s41927-022-00274-y

**Published:** 2022-08-02

**Authors:** Thomas E. Bolhuis, Diane Marsman, Frank H. J. van den Hoogen, Alfons A. den Broeder, Nathan den Broeder, Aatke van der Maas

**Affiliations:** 1 Department of Rheumatology, Sint Maartenskliniek, Ubbergen, The Netherlands; 2 Radboud Institute for Health Sciences, Radboud University Medical Center, Nijmegen, The Netherlands; 3 Department of Rheumatology, Radboudumc, Nijmegen, The Netherlands

**Keywords:** Polymyalgia rheumatica, Epidemiology, Prognosis, Prediction, Risk factors, Relapse

## Abstract

**Background:**

To develop and assess a prediction model for polymyalgia rheumatica (PMR) relapse within the first year of glucocorticoid (GC) treatment.

**Methods:**

A retrospective PMR cohort (clinical diagnosis) from a rheumatology department was used. All visits > 30 days after starting GC treatment and with > 2.5 mg/day oral prednisolone were used as potential relapse visits. Often used relapse criteria (1) rheumatologist judgement, (2) treatment intensification-based relapse) were assessed for agreement in this cohort. The proportion of patients with treatment-based relapse within 1 and 2 years of treatment and the relapse incidence rate were used to assess unadjusted associations with candidate predictors using logistic and Poisson regression respectively. After using a multiple imputation method, a multivariable model was developed and assessed to predict the occurrence (yes/no) of relapse within the first year of treatment.

**Results:**

Data from 417 patients was used. Relapse occurred at 399 and 321 (of 2422) visits based on the rheumatologist judgement- and treatment-based criteria respectively, with low to moderate agreement between the two (87% (95% CI 0.86–0.88), with κ = 0.49 (95% CI 0.44–0.54)). Treatment-based relapse within the first two years was significantly associated with CRP, ESR, and pre-treatment symptom duration, and incidence rate with only CRP and ESR. A model to predict treatment intensification within the first year of treatment was developed using sex, medical history of cardiovascular disease and malignancies, pre-treatment symptom duration, ESR, and Hb, with an AUC of 0.60–0.65.

**Conclusion:**

PMR relapse occurs frequently, although commonly used criteria only show moderate agreement, underlining the importance of a uniform definition and criteria of a PMR specific relapse. A model to predict treatment intensification was developed using practical predictors, although its performance was modest.

**Supplementary Information:**

The online version contains supplementary material available at 10.1186/s41927-022-00274-y.

## Background

Polymyalgia rheumatica (PMR) is an inflammatory rheumatic disease characterized by complaints of the neck, shoulder and hip girdle and a rise in acute phase reactants (APR) [1]. The mainstay of treatment is glucocorticoids (GC), which are tapered after achieving remission to reduce frequently occurring adverse events [2]. However, during this tapering, disease relapses occur in up to 55% of patients, necessitating an increase of therapy and increasing risk of adverse events [3–5]. Therefore, prediction of relapse would help identify patients benefitting more from intense early treatment—like starting a GC sparing agent or faster tapering.

However, the value of predictors for PMR relapse is still unclear, as studies show conflicting results [3, 5–10]. This may be due to the relatively limited number of patients and relapses or due to the heterogeinity of relapse definitions and criteria, which hinders comparison between studies [8]. Moreover, a multivariable prediction model combining risk factors, which may assess the overall combined value of predictors, has not been implemented.

Using a prediction model to estimate a patient’s risk of PMR relapse at the start of therapy may help in the decision of starting disease-modifying antirheumatic drugs (DMARDs) at onset or taper GC more judiciously. In addition, such a model may help stratify patients by prognosis for clinical studies. Therefore, we set out to develop and assess a prediction model for relapse within the first year of treatment. Furthermore, we will describe our choices leading to two different criteria for relapse and assess agreement between these criteria.

## Methods

### Design and setting

Our goal was to develop and assess a prediction model for relapse within the first year of treatment, based on data from a routine care retrospective cohort of PMR patients visiting the outpatient rheumatology department of the Sint Maartenskliniek, Netherlands, between April 2008 and January 2017. Since multiple criteria have been used to measure relapse, we will explain our choices leading to two different criteria and assess agreement between these. By doing so, we hope to gain insight in the degree of heterogeneity in this outcome and improve comparison between our study and future studies. Furthermore, since we wanted to identify patients who will benefit most from intense early treatment (e.g., starting a DMARD), we chose one criterion for relapse which we think is most closely related to treatment, and thus has most implications, for studying association and modelling. The transparent reporting of a multivariable prediction model for individual prognosis or diagnosis (TRIPOD) guideline was used for reporting purposes, and the TRIPOD checklist is shown in Additional file 1 [11].

### Study population and data collection

Records of patients with a clinical PMR diagnosis (electronically registered by treating physicians) were reviewed for cohort eligibility. Patients were excluded if an alternative diagnosis was more likely (judged initially by the treating rheumatologist and retrospectively reviewed by the research physician), if follow-up was less than nine months, or if patients were deceased. Patients were treated and assessed in accordance with international recommendations [2].

Baseline assessment was the first visit that prednisolone treatment started and follow-up thereafter ended either at the end of the study period (January 2017) or when lost to follow-up. Follow-up visits more than 90 days after oral prednisolone treatment ended were also excluded. We chose to do this, since recurrences after GC free remission have less treatment implications and less impact on GC-related adverse events when compared with relapses during GC tapering [2]. Furthermore, recurrences after treatment cessation may differ pathophysiologically when compared with relapses [2, 12].

### Primary outcome: relapse

Based on previous studies, we pragmatically chose a period of initial response of 30 days after starting prednisolone during which no relapse could occur, so each patient had a period of potential remission before a relapse [4, 13]. We also only used visits with > 2.5 mg/day oral prednisolone to exclude relapses at relatively low-dose prednisolone, since these have fewer implications for treatment and treatment related adverse events. Additionally, this may help exclude pragmatic prednisolone dose raising or maintenance due to concomitant or mimicking diseases flaring at this low dose in regular care. After these exclusions, we chose two approaches identifying a PMR relapse, based on The Outcome Measures in Rheumatology (OMERACT) definition for a rheumatoid arthritis flare [14].

1. A rheumatologist judgement-based relapse consisted of either (a) judgement of the treating rheumatologist that a patient is not in remission during a visit, or (b) judgement of the treating rheumatologist and/or patient that a patient had a relapse between visits, both of which in combination with no RJ at the previous visit.
2. A treatment intensification-based relapse consisted of either (a) an increase in prednisolone dose (between visits) or rheumatologist advice to increase prednisolone dose, (b) starting/increasing dose of a DMARD due to treatment inefficacy, or (c) addition of a local or intra-muscular GC injection, all in combination with no TI at the previous visit.

We chose the treatment based criteria to study associations and for further prediction modelling, since this is a more objective and pragmatical outcome closely related to treatment. Therefore, prediction of TI based relapse may have the most treatment implications.

### Candidate predictors

Candidate predictors for treatment intensification-based relapse were measured at baseline, and thus before GC treatment started. The following predictors were assessed: (1) age, (2) sex, (3) medical history of cardiovascular disease, i.e. a previous myocardial infarction, angina, cerebrovascular events, peripheral arterial disease, heart failure, or thoracic aortic aneurysm, (4) medical history of malignancies, (5) smoking defined as either never, stopped, or current smoker, (6) pre-treatment symptom duration in months, (7) clinical disease severity based on presence of pain/stiffness and movement restriction at both shoulder- and hip girdles, resulting in a 0–8 range sum score, (8) presence of clinically diagnosed peripheral arthritis, (9) presence of systemic symptoms, based on the presence of either fever, cold shivering, night sweats, unexplained weight loss, or unexplained fatigue, (10) C-reactive protein (CRP) level in mg/L determined by the chemical analyzer Olympus type AU400, (11) Erythrocyte Sedimentation Rate (ESR) level in mm/h determined by the 30-min automated and extrapolated version of the Westergren method, and (12) hemoglobin (Hb) level in mmol/L. ESR and CRP were divided by a factor 10 for use as predictors to improve coefficient interpretability.

### Sample size

Previous studies reported relapse percentages between 27.6 and 55% within the first (five) year(s) of treatment [3–5]. Therefore, with the 450 patient cohort, approximately 33% of patients having an event (relapse) within the first year of treatment, and a rule of thumb of 10 events per variable, we estimate 15 predictors could be included [15].

### Analysis

STATA/IC version 13.1 for Windows was used for all statistical analysis. Descriptive statistics were examined as appropriate. Numbers and reason for exclusion were recorded to ensure internal validity. Relapse on a visit level was identified for both criteria, and agreement was assessed using percentage of agreement and Cohen’s kappa. Relapse cumulative incidence (proportion of patients) after one and two year(s) of treatment and relapse incidence rate (IR) per patient per year was assessed for both rheumatologist judgement and treatment intensification relapse.

Unadjusted associations between the proportion of patients with a treatment intensification-based relapse and predictors was assessed using logistic regression. Unadjusted associations between the incidence rate of treatment intensification-based relapse and predictors was assessed using Poisson regression. Results were displayed using odds ratios (OR), incidence rate ratios (IRR), 95% confidence intervals in brackets, and we considered *p* values < 0.05 as significant.

After using a multiple imputation method, a multivariable model was established to predict the occurrence (yes/no) of relapse within the first year of treatment and this model’s predictive performance and internal validity was assessed thereafter. Furthermore, a sensitivity analysis was performed in which only patient fulfilling EULAR/ACR core classification criteria (without scoring scale) were used to develop and assess a multivariable model. Further detail on model development and assessment is given in Additional file 2.

## Results

**Fig. 1:**
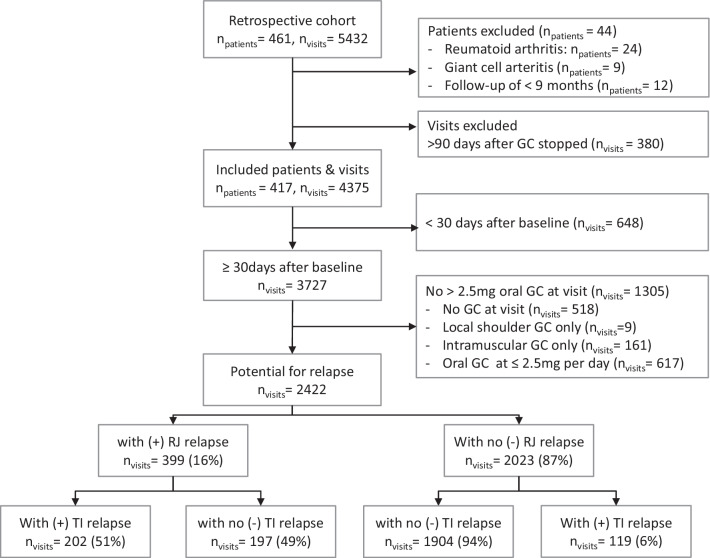
Flow chart of patients and visits with a (potential) relapse based on rheumatologist judgement (RJ) and treatment intensification (TI)

From the cohort, 417 patients with 4375 visits were included (Fig. 1). There were 2422 visits that met criteria for a potential relapse (≥ 30 days after baseline and treated with > 2.5 mg oral prednisolone). Details of the cohort at baseline are shown in Table 1: age was comparable to previous PMR studies, although sex differed somewhat, with a lower percentage of females [3, 5, 12, 16, 17]. A total of 329 (79%) patients met 2012 European League Against Rheumatism/American College of Rheumatology (EULAR/ACR) core classification criteria and 353 (90%) had raised APR from those with APR available [18]. There were missing values (n; %) for smoking (81; 19%), pre-treatment symptom duration (6; 1%), CRP (53; 13%), ESR (21; 5%), and Hb (100; 24%).

**Table 1:** Cohort description at baseline

Variables	Total n = 417	(100%)
Age in years, mean (SD)	66.4	(8.8)
Sex female	233	(55.9%)
*Positive medical history for*		
Malignancy	51	(12.2%)
cardiovascular disease	59	(14.1%)
*Smoking*^*1*^		
No	197	(47.2%)
Stopped	87	(20.9%)
Active	52	(12.5%)
Pre-treatment symptom treatment in weeks, mean (SD)^2^	14	(13.7)
Presence of systemic symptoms	182	(43.6%)
(Suspected) presence of peripheral arthritis	64	(15.3%)
*Shoulder pain/stiffness*		
No	11	(2.6%)
Unilateral	16	(3.8%)
Bilateral	390	(93.5%)
*Hip pain/stiffness*		
No	39	(9.4%)
Unilateral	10	(2.4%)
Bilateral	368	(88.2%)
*Movement restriction shoulders*		
No	222	(53.2%)
Unilateral	26	(6.2%)
Bilateral	169	(40.5%)
*Movement restriction hips*		
No	325	(77.9%)
Unilateral	26	(6.2%)
Bilateral	66	(15.8%)
CRP in mg/L, median (IQR)^3^	29	(15–52.5)
ESR in mm/h, median (IQR)^4^	37	(26–51)
Hb in mmol/L, median (IQR)^5^	8.2	(7.6–8.6)

SD, standard deviation; n, number; IQR, interquartile range; CRP, C-Reactive Protein; ESR, erythrocyte sedimentation rate; Hb, hemoglobin

^1^n = 336, ^2^n = 411, ^3^n = 364, ^4^n = 396, ^5^n = 317

There were 399 and 321 relapse visits based on rheumatologist judgement and treatment intensification respectively. Agreement between criteria was 87% and κ = 0.49 (95% CI 0.44–0.54). Therefore, agreement was low to moderate based on kappa, although not on percentage. A reason for the low kappa when compared to percentage agreement may be the relatively high predicted probability of agreement due to chance for visits without a relapse (72%). Based on rheumatologist judgement and treatment intensification, relapse cumulative incidence was 156 (37%) and 133 (32%) after one year, and 202 (48%) and 183 (44%) after two years of treatment, respectively. Furthermore, agreement between criteria for cumulative incidence after one and two years of treatment was 83% (95% CI 0.79–0.87) with κ = 0.63 (95% CI 0.55–0.71) and 84% (95% CI 0.80–0.87) with κ = 0.68 (95% CI 0.61–0.75) respectively. Total follow-up duration was 911 a patient years, resulting in a rheumatologist judgement and treatment intensification- based relapse IR of 0.44 and 0.35 (per patient per year) respectively.

The treatment intensification criterion was used to study univariable associations with potential predictors (Table 2). Relapse cumulative incidence after both one, and two, year(s) of treatment was significantly associated with pre-treatment symptom duration, CRP, and ESR. For example, the odds of having a relapse, after both 1 and 2 years of treatment, is 8% lower per week of pre-treatment symptom duration. Total relapse IR per patient per year was significantly associated with CRP and ESR, although, interestingly, not with pre-treatment symptom duration. For example, the number of relapses per patient year was 4% higher per 10 mg/L increase in CRP.

**Table 2:** Univariable associations between predictors and different outcomes of relapse

Predictors	Relapse within year 0–1				Relapse within year 0–2				Relapses per patient per year			
	OR	95% CI		*p*	OR	95% CI		*p*	IRR	95% CI		*p*
Age (years)	1.00	0.98	0.92	0.92	1.00	0.76	1.02	0.76	1.00	0.99	1.01	0.87
Sex (reference category: male)	1.42	0.930	0.11	0.11	1.36	0.12	2.01	0.12	1.00	0.78	1.26	0.96
*Positive medical history (reference category: no)*												
Inflammatory disease	1.20	0.76	0.43	0.43	1.01	0.96	1.55	0.96	1.21	0.94	1.58	0.14
Malignancy	0.62	0.32	0.17	0.17	0.96	0.68	1.60	0.68	0.91	0.62	1.34	0.63
Cardiovascular disease	1.70	0.97	0.06	0.06	1.76	0.05	3.07	0.05	1.20	0.89	1.64	0.24
*Smoking (reference category: no)*^1^												
Stopped	0.87	0.50	0.61	0.61	1.18	0.52	1.96	0.52	1.29	0.96	1.74	0.09
Yes	0.82	0.42	0.57	0.57	1.00	0.99	1.86	0.99	0.97	0.67	1.40	0.86
Symptom duration before baseline in months^1^	***0.92***	***0.85***	***0.03***	***0.03***	***0.92***	***0.02***	***0.99***	***0.02***	0.96	0.92	1.00	0.09
Clinical disease severity (score 0–8)	0.93	0.81	0.29	0.29	0.92	0.20	1.05	0.20	0.94	0.86	1.01	0.10
(Suspected) presence of peripheral arthritis (reference category: no)	0.74	0.41	0.32	0.32	0.68	0.17	1.18	0.17	0.79	0.54	1.15	0.22
Presence of systemic symptoms (reference category: no)	0.80	0.52	0.29	0.29	0.86	0.44	1.27	0.44	1.00	0.79	1.27	0.98
CRP per 10 mg/L^2^	***1.08***	***1.01***	***0.02***	***0.02***	***1.09***	***0.01***	***1.16***	***0.01***	***1.04***	***1.01***	***1.08***	***0.03***
ESR per 10 mm/h^3^	***1.20***	***1.08***	***0.00***	***0.00***	***1.22***	***0.00***	***1.35***	***0.00***	***1.09***	***1.03***	***1.15***	***0.00***
Hb per mmol/L^4^	1.03	0.75	0.88	0.88	0.99	0.94	1.32	0.94	1.03	0.86	1.25	0.73

n, number; CRP, C-Reactive Protein; ESR, erythrocyte sedimentation rate; Hb, hemoglobin; APR, acute phase reactants; OR, odds ratio; CI, confidence interval; IRR, incidence rate ratio

Bold, italic results show significance at a *p* value < 0.05. ^1^n = 411, ^2^n = 364, ^3^n = 396, ^4^n = 317

A multivariable prediction model for relapse within the first year of treatment was established with the variables sex, medical history of cardiovascular disease, medical history of malignancies, pre-treatment symptom duration, ESR serum level, and Hb serum level. Although this model was stable, its predictive performance was only modest (e.g., the concordance statistic or area under the curve ranged between 0.60 and 0.65). A sensitivity analysis, using only patients fulfilling EULAR/ACR core criteria, resulted in a model with the variables sex, medical history of cardiovascular disease, pre-treatment symptom duration, ESR serum level, and Hb serum level. Medical history of malignancies was included in only 40% of imputation sets and thus not included in the final model. However, (optimism corrected) performance sensitivity analysis model was comparable to model of the full population. Further detail on the model, its performance, and an example application is given in Additional file 2.

## Discussion

We found a high number of relapses, although agreement between different criteria was limited, with a lower amount of treatment intensification compared to clinical relapse. The number of relapses is in line with previous studies, although agreement between criteria has not been studied before [3–5].

The limited agreement between both relapse definitions may suggest treatment intensification is avoided, even though a physician may suspect a relapse to be present. This may be because of the expected exacerbating or development of concomitant disorders, as PMR is a disease of the elderly, and therefore patients and physicians may choose self-management over treatment intensification [16, 19]. Indeed, the higher agreement after one and two years compared visits, may support the hypothesis that a part of patients and physicians wait before ultimately intensifying treatment. Another reason may be, a PMR specific relapse may be difficult to discern from a flare up due to a concomitant disorder, especially when relying on non-specific symptoms [3, 5, 20, 21]. Discerning a relapse may therefore be facilitated by a composite measure—like the PMR-activity score—although further clinimetric research is required before one can be recommended [20, 22–27].

We found an unexpected association with relapse and used multiple predictors to develop a multivariable model, although with relatively modest performance. Increased pre-treatment symptom duration was associated with lower odds of relapse within 1–2 years after start of treatment, but not with number of relapses per year. This may be due to residual confounding or lead time bias, although the effect persisted when corrected for level of serum inflammatory markers, age and sex (not shown). Furthermore, it may be that the rate of relapse is highest in the first (two) years of PMR symptoms [9], and therefore lead time could explain why PMR patients with longer existing symptoms before treatment may have a head start to a relatively self-limiting disease course [28]. An important reason for the potentially limited performance of the prediction model may be the exclusion of some strong predictors that are not either not routinely available (e.g., baseline angiopoietin-2 levels), or not available before treatment (e.g., GC tapering speed) [4, 12].

Some limitations and strengths should be noted. A limitation of this study with regards to diagnosing a relapse is its retrospective design, although this limitation is found more often in studies on relapse [4, 16]. Furthermore, this design excluded health status before treatment based on EQ-5D as a potential predictor [29]. A strength of this study is the clear description of relapse and use of pragmatic predictors, and how this relates to our goal of predicting treatment intensification at the start of therapy. Furthermore, our large sample size and use of all data through multiple imputation resulted in a valid and stable model.

## Conclusion

This retrospective study emphasizes the lack of a uniform definition of relapse and a current inability to stratify patients by prognosis. Therefore, focus should be on reaching consensus on a definition and criteria—preferably based on a composite measure—for relapse. Thereafter, focus should shift on developing a model to identify patients with worse or better prognosis who might benefit more from more intense treatment (e.g., DMARDs) or faster tapering. Furthermore, to ease implementation, practical predictors that are both widely available and available at the start of treatment are preferable, especially considering a large proportion of patients is treated in primary care.

## Supplementary Information

**Additional file 1**. TRIPOD checklist

**Additional file 2**. Elaboration on model development and assessment

## Data Availability

The datasets used and analyzed in the study are available from the corresponding author on reasonable request.

## Abbreviations

PMR: Polymyalgia rheumatica
APR: Acute phase reactants
GC: Glucocorticoids
DMARDs: Disease-modifying antirheumatic drugs
TRIPOD: Transparent reporting of a multivariable prediction model for individual prognosis or diagnosis
OMERACT: Outcome Measures in Rheumatology
RJ: Rheumatologist judgement-based relapse
TI: Treatment intensification-based relapse
CRP: C-reactive protein
ESR: Erythrocyte Sedimentation Rate
Hb: Hemoglobin
OR: Odds ratio
IR: Incidence ratio
IRR: Incidence rate ratio
EULAR/ACR: European League Against Rheumatism/American College of Rheumatology
