# Supplementary material for: (Dis)agreement of polymyalgia rheumatica relapse criteria, and prediction of relapse in a retrospective cohort
Source: BMC Rheumatol. 2022 Aug 2;6:45. doi: 10.1186/s41927-022-00274-y (PMC9344672; doi:10.1186/s41927-022-00274-y)
Supplement: Supplementary file 2 — Additional file 2. Elaboration on model development and assessment [file 41927_2022_274_MOESM2_ESM.docx]

# Additional file 2: Elaboration on model development and assessment

## Methods

Predictors.

Predictors were assessed with regards to collinearity using a correlation matrix and distribution using histograms. Thereafter, missing values were assessed with regards to amount and randomness using visualization (e.g. forest plots) and Little’s test for missing completely at random (MCAR) (1). We used multivariate imputation using chained equations (MICE) to impute 100 data sets, with a predictive mean matching model with 10 nearest neighbors and 20 burn-ins per imputation (2, 3). All predictor variables and the outcome relapse within the first year of treatment were used in the model to impute data sets (4). Five complete data sets were assessed with regards to plausibility.

Development.

We used a backwards stepwise selection procedure for fitting a logistic regression model in all imputation sets (separately), using a conservative cut-off for removal of p > 0.20 (5, 6). Predictors included in > 50% of the imputation sets were chosen for a final model (7, 8).

Performance.

We assessed predictive performance for each model in its corresponding imputation set (9). The Brier score was used as a measure for overall performance (4, 10). The concordance statistic (c-statistic) was used to measure discrimination. A calibration plot was made to assess calibration by dividing the predicted probability in 10 groups, calculating the mean observed and predicted probability for these groups, and plotting a point in a graph (y=mean(predicted) and x=mean(observed) per group. A linear regression line was derived from the 10 points with the slope corresponding to the c-slope and the intercept corresponding to the c-intercept.

Internal validation.

We used bootstrapping to internally validate our model’s predictive performance using 100 bootstrap samples per imputation set (4).

Shrinkage.

Model coefficients were adjusted with a shrinkage factor to account for potential testimation bias as a consequence of the predictor selection process. The shrinkage factor was obtained using the slope of the linear predictor we derived from the 100 bootstrap samples per imputation set (4). The shrinkage adjusted model was applied to its corresponding imputation set and predictive performance was assessed.

Pooling of results.

Pooling of model parameters (i.e., coefficients and SE) and predictive performance of the 100 imputation sets was done using Rubin's Rules and medians (ranges) respectively (3).

Sensitivity analysis.

As sensitivity analysis, previous steps for model development and assessment were repeated for the 329 (79%) patients who met 2012 European League Against Rheumatism/American College of Rheumatology (EULAR/ACR) core classification criteria (11).

## Results

Model development.

Due to missing values, a lack of a strong association, and imputation issues due to a lack of hierarchical ordering, smoking was dropped as predictor. Relapse occurrence within the first year of treatment had 11.2 events per variable based on the 12 predictors left. For complete case analysis there were 293 patients with 89 (30.4%) events. Missings were hypothesized to be missing at random after assessment, with Little’s test for MCAR supporting MCAR (p = 0.36) (1). Consequently, MICE was used to create 100 complete datasets. Predictors included in 100% of the (stepwise) models were sex, medical history of cardiovascular disease, medical history of malignancy, symptom duration before treatment, ESR, and Hb. Moreover, the presence of predictors in 100% of the models indicated a stable model.

Model assessment and internal validation.

Model parameters and predictive performance was assessed in each imputation set and pooled as shown in **table 1** and **table 2** respectively. General performance hardly changed after correction for optimism, with a Brier score increasing from 0.20 to 0.21 (**table 2**). Calibration, especially calibration-slope, substantially decreased after correction for optimism, with a slope and intercept going from 0.98 and 0.00 to 0.86 and 0.04 respectively. Discrimination, although modest, hardly changed after correction for optimism, going from 0.65 to 0.63.

| **Table 1.** Prediction model parameters | | | | |
| --- | --- | --- | --- | --- |
|  | **Original model** | | **Shrunk model#** | |
| **Predictor** | **β†** | **(SE)†** | **β†** | **OR** |
| Female sex | 0.73 | 0.26 | 0.62 | 1.86 |
| Medical history of malignancy | -0.54 | 0.36 | -0.46 | 0.63 |
| Medical history of cardiovascular disease | 0.76 | 0.31 | 0.65 | 1.92 |
| Symptom duration before GC, per month | -0.02 | 0.01 | -0.02 | 0.98 |
| ESR, per 10 mm/hour | 0.03 | 0.01 | 0.02 | 1.02 |
| Hb, per mmol/L | 0.48 | 0.19 | 0.40 | 1.49 |
| Intercept/constant | -5.95 | 1.83 | -5.18 |  |
| Abbreviations: ESR = erythrocyte sedimentation rate; Hb = hemoglobin; β = regression coefficients.  *Note.* †Model parameters were pooled using Rubin’s Rules. Examples for calculating the predicted probability of relapse are given in the supplement. # The shrunk model was derived from the original model after multiplying regression coefficients with a shrinkage factor (per specific imputation set). | | | | |

| **Table 2*.*** Predictive performance | | | | |
| --- | --- | --- | --- | --- |
| **Performance measure** | **Original**  **Model** | **Optimism** | **Optimism**  **corrected** | **Shrunk**  **Model†** |
| Brier score | 0.20  (0.20-0.20) | -0.01  (-0.01-0.00) | 0.21  (0.20-0.21) | 0.20  (0.20-0.20) |
| Calibration slope | 0.98  (0.92-1.06) | 0.12  (0.08-0.16) | 0.86  (0.77-0.94) | 1.13  (1.05-1.23) |
| Calibration intercept | 0.00  (-0.02-0.02) | -0.04  (-0.05-(-0.02)) | 0.04  (0.02-0.07) | -0.04  (-0.08-(-0.02)) |
| Concordance statistic | 0.65  (0.64-0.67) | 0.02  (0.02-0.03) | 0.63  (0.61-0.65) | 0.65  (0.64-0.67) |
| *Note.* Optimism was calculated using 100 bootstrap samples per imputed data set. Model performance was pooled over the 100 imputation sets using medians (ranges). † Shrinkage factor was obtained using the linear predictor slope from the 100 bootstrap samples in each (separate) imputation set. | | | | |

Sensitivity analysis.

Predictors included in the (stepwise) models were sex, medical history of cardiovascular disease, symptom duration before treatment, and ESR, and Hb. However, in this sensitivity analysis, medical history of malignancy was only included in 40% of models and, therefore, not included ultimately indicating a less stable model. Th sensitivity analysis model parameters and predictive performance were also assessed in each imputation set and pooled as shown in **table 3** and **table 4** respectively. Overall, performance and optimism corrected performance was compared to the full sample analysis.

| **Table 3.** Sensitivity analysis prediction model parameters | | | | |
| --- | --- | --- | --- | --- |
|  | **Original model** | | **Shrunk model#** | |
| **Predictor** | **β†** | **(SE)†** | **β†** | **OR** |
| Female sex | 0.74 | 0.28 | 0.62 | 1.86 |
| Medical history of cardiovascular disease | 0.66 | 0.35 | 0.55 | 1.73 |
| Symptom duration before GC, per month | -0.09 | 0.04 | -0.08 | 0.92 |
| ESR, per 10 mm/hour | 0.27 | 0.07 | 0.22 | 1.25 |
| Hb, per mmol/L | 0.51 | 0.22 | 0.42 | 1.52 |
| Intercept/constant | -6.14 | 2.03 | -5.22 |  |
| Abbreviations: ESR = erythrocyte sedimentation rate; Hb = hemoglobin; β = regression coefficients.  *Note.* †Model parameters were pooled using Rubin’s Rules. Examples for calculating the predicted probability of relapse are given in the supplement. # The shrunk model was derived from the original model after multiplying regression coefficients with a shrinkage factor (per specific imputation set). | | | | |

| **Table 4*.*** Sensitivity analysis predictive performance | | | | |
| --- | --- | --- | --- | --- |
| **Performance measure** | **Original**  **Model** | **Optimism** | **Optimism**  **corrected** | **Shrunk**  **Model†** |
| Brier score | 0.21  (0.20-0.21) | -0.01  (-0.01-0.01) | 0.22  (0.21-0.22) | 0.21  (0.20-0.21) |
| Calibration slope | 0.96  (0.90-1.04) | 0.14  (0.09-0.18) | 0.83  (0.74-0.90) | 1.13  (1.06-1.25) |
| Calibration intercept | 0.01  (-0.01-0.04) | -0.04  (-0.06-(-0.03)) | 0.06  (0.04-0.09) | -0.04  (-0.09-(-0.02)) |
| Concordance statistic | 0.64  (0.62-0.67) | 0.03  (0.02-0.03) | 0.61  (0.59-0.63) | 0.64  (0.62-0.67) |
| *Note.* Optimism was calculated using 100 bootstrap samples per imputed data set. Model performance was pooled over the 100 imputation sets using medians (ranges). † Shrinkage factor was obtained using the linear predictor slope from the 100 bootstrap samples in each (separate) imputation set. | | | | |

## Application

Odds(relapse) = exp (βcons + β*sex + + β*malignancy β*cardiovascular + β*duration + β*ESR + β*Hb)

Odds(relapse) = exp (–5.18 + 0.62*sex + (-0.46)*malignancy + 0.65*cardiovascular + (-0.02)*duration + 0.02*ESR + 0.40*Hb)

Where:

Sex: 1 = female and 0 = male

Malignancy: 1 = positive medical history and 0 = negative

Cardiovascular: 1 = positive medical history and 0 = negative

Duration: amount of weeks of symptoms before GC treatment

ESR: ESR in 10mm/hour

Hb: hemoglobin level in mmol/L

*Example case 1:* A man (0), with no malignancies in his medical history (0), a history of cardiovascular disease (1), 2 months of symptoms before treatment (2), baseline ESR of 40mm/h (4), and baseline Hb of 9 mmol/L (9)

Odds(relapse) = exp (–5.18 + 0.62*0 + (-0.46)*0 + 0.65* 1 + (-0.02)* 2 + 0.02*4 + 0.40*9) = exp(-0.92)

probability(relapse) = odds/(odds+1) = exp(-0.92) /(exp(-0.92) +1) = 0.47

*Example case* 2: A woman (1), with 1 month of symptoms before treatment (1), baseline ESR of 70 mm/h (7), and baseline Hb of 8 mmol/L (8)

Odds(relapse) = exp (0.11) = 1.12 and Probability(relapse) = 0.5

## References

1. Li C. Little's Test of Missing Completely at Random. The Stata Journal. 2013;13(4):795-809.

2. Morris TP, White IR, Royston P. Tuning multiple imputation by predictive mean matching and local residual draws. BMC Med Res Methodol. 2014;14(1):75.

3. Buuren S. Flexible Imputation of Missing Data. 2nd ed: Chapman and Hall/CRC; 2018.

4. Steyerberg E. Clinical Prediction Models: A Practical Approach to Development, Validation, and Updating: New York: Springer; 2009.

5. Ambler G, Brady AR, Royston P. Simplifying a prognostic model: a simulation study based on clinical data. Stat Med. 2002;21(24):3803-22.

6. Royston P, Moons KGM, Altman DG, Vergouwe Y. Prognosis and prognostic research: Developing a prognostic model. BMJ. 2009;338:b604.

7. Heymans MW, van Buuren S, Knol DL, van Mechelen W, de Vet HCW. Variable selection under multiple imputation using the bootstrap in a prognostic study. BMC Med Res Methodol. 2007;7:33-.

8. Wood AM, White IR, Royston P. How should variable selection be performed with multiply imputed data? Stat Med. 2008;27(17):3227-46.

9. Marshall A, Altman DG, Holder RL, Royston P. Combining estimates of interest in prognostic modelling studies after multiple imputation: current practice and guidelines. BMC Med Res Methodol. 2009;9:57-.

10. Moons KGM, Altman DG, Reitsma JB, Ioannidis JPA, Macaskill P, Steyerberg EW, et al. Transparent Reporting of a multivariable prediction model for Individual Prognosis or Diagnosis (TRIPOD): explanation and elaboration. Ann Intern Med. 2015;162(1):W1-73.

11. Dasgupta B, Cimmino MA, Maradit-Kremers H, Schmidt WA, Schirmer M, Salvarani C, et al. 2012 Provisional classification criteria for polymyalgia rheumatica: A European League Against Rheumatism/American College of Rheumatology collaborative initiative. Ann Rheum Dis. 2012;71(4):484-92.
